# Retail sector distribution chains for malaria treatment in the developing world: a review of the literature

**DOI:** 10.1186/1475-2875-9-50

**Published:** 2010-02-11

**Authors:** Edith Patouillard, Kara G Hanson, Catherine A Goodman

**Affiliations:** 1 London School of Hygiene and Tropical Medicine, Keppel Street, London, UK; 2 Kenya Medical Research Institute - Wellcome Trust Research Programme, Nairobi, Kenya

## Abstract

**Background:**

In many low-income countries, the retail sector plays an important role in the treatment of malaria and is increasingly being considered as a channel for improving medicine availability. Retailers are the last link in a distribution chain and their supply sources are likely to have an important influence on the availability, quality and price of malaria treatment. This article presents the findings of a systematic literature review on the retail sector distribution chain for malaria treatment in low and middle-income countries.

**Methods:**

Publication databases were searched using key terms relevant to the distribution chain serving all types of anti-malarial retailers. Organizations involved in malaria treatment and distribution chain related activities were contacted to identify unpublished studies.

**Results:**

A total of 32 references distributed across 12 developing countries were identified. The distribution chain had a pyramid shape with numerous suppliers at the bottom and fewer at the top. The chain supplying rural and less-formal outlets was made of more levels than that serving urban and more formal outlets. Wholesale markets tended to be relatively concentrated, especially at the top of the chain where few importers accounted for most of the anti-malarial volumes sold. Wholesale price mark-ups varied across chain levels, ranging from 27% to 99% at the top of the chain, 8% at intermediate level (one study only) and 2% to 67% at the level supplying retailers directly. Retail mark-ups tended to be higher, and varied across outlet types, ranging from 3% to 566% in pharmacies, 29% to 669% in drug shops and 100% to 233% in general shops. Information on pricing determinants was very limited.

**Conclusions:**

Evidence on the distribution chain for retail sector malaria treatment was mainly descriptive and lacked representative data on a national scale. These are important limitations in the advent of the Affordable Medicine Facility for Malaria, which aims to increase consumer access to artemisinin-based combination therapy (ACT), through a subsidy introduced at the top of the distribution chain. This review calls for rigorous distribution chain analysis, notably on the factors that influence ACT availability and prices in order to contribute to efforts towards improved access to effective malaria treatment.

## Background

In many low- and middle-income countries, the retail sector plays an important role in the provision of malaria treatment [1-14]. For example, it was the first source of care for around 45% of households seeking malaria treatment across four communities in Enugu State, Nigeria [15] and in three rural districts of Tanzania nearly 40% of all anti-malarial volumes were dispensed within the retail sector [16]. Retail providers tend to operate closer to homes [15,17-19] and offer a more reliable and wider range of drugs than public health providers [2,11,14,18-20], sometimes at lower costs [14,21-23].

The market for anti-malarial drugs includes artemisinin-based combination therapy (ACT), which is the most effective drug regimen and the official first-line treatment in most developing countries, non-artemisinin drugs, some of which were recommended before the ACT era (e.g. chloroquine, amodiaquine, sulphadoxine-pyrimethamine and quinine), and artemisinin monotherapies. These three product types are available under different formulations including tablets, suppositories, suspensions, syrups and liquid injectables. Some are sold under their proprietary names, and referred to as innovator brands when they are products patented by their originators, or branded generics in the case of generic versions of innovator products marketed under a different name. Others are sold as unbranded generics without a proprietary name.

Within the retail market, these products are sold by a wide range of providers whose characteristics vary substantially across settings. Providers can be pharmacies, drug shops, grocery stores, market stalls or itinerant hawkers. In East and West Africa, drug shops that specialize in handling drugs play a major role, such as in Tanzania where they accounted for 88% of retail sector anti-malarial sales volumes [16]. Mobile vendors are common in West Africa, but are rarely found in East and Southern Africa [24]. Outlets staffed by trained pharmacists are rare in all countries [17,25], and concentrated in urban areas, whilst drug shops can be found in both urban and more densely populated rural areas. Finally, general shops that sell drugs alongside household goods are often the only medicine retailers in more remote rural villages.

Pharmacies are generally authorized to stock both prescription-only drugs and over-the-counter (OTC) products, while other outlets can only sell OTC drugs, although in practice some illegally stock prescription-only medicines [24]. Whilst anti-malarial drug availability is relatively high in the retail sector [19,25-30], the range of anti-malarials is generally lower in outlets which are more remote or have less qualified staff [19,25,28,31]. ACT is rarely available outside facilities and pharmacies because of their high price relative to older, less effective alternatives. For example, in six districts of Zambia, ACT accounted for only 7% of all anti-malarials sold in the retail sector [33] and in Tanzania, the old monotherapy sulphadoxine-pyrimethamine (SP) was the most commonly retailed anti-malarial, followed by artemisinin monotherapies [34]. The availability of artemisinin monotherapies is highly variable, but a major cause of concern as their use is likely to contribute to the development of artemisinin resistance [35].

Other concerns around the quality of care provided in the retail sector relate to retailers' lack of qualifications, poor knowledge of drugs and dosages [36-39], and stocking of unregistered [28,31] and sometimes substandard or counterfeit drugs [6,19,40-44]. Although care provided by pharmacies is far from perfect [45,46], most of these concerns are directed to non-pharmacy outlets. Drug shop staff are rarely qualified pharmacists [47], having at best a basic nursing background [24,26] or sometimes just secondary education [47]. General retailers have even fewer qualifications and some are illiterate [18,20].

These drug retailers are the last link in a chain of suppliers and their practices are likely to be heavily influenced by what happens further up the distribution chain. Retail availability, for instance, will be affected by which products are available from suppliers, the marketing strategies used to promote certain drugs, and the registration of drugs and regulation of providers further up the chain. Retail prices will be influenced by wholesale prices, and the cost of obtaining and storing goods. Retail quality will be determined by how products have been handled and stored higher up the chain. In turn, the behaviour of suppliers in the chain will be influenced by the nature of competition and regulation that they face.

Understanding the distribution chain for anti-malarials is, therefore, crucial in designing interventions to improve retail sector care. This is of particular importance in the light of the implementation of the Affordable Medicines Facility for Malaria (AMFm), which will rely on existing distribution chains to deliver heavily subsidized ACT to consumers. This article aims to support such initiatives by summarizing the current state of knowledge on the retail sector distribution chain for malaria treatment in low- and middle-income countries.

## Methods

### Scope of the review

**Figure 1:**
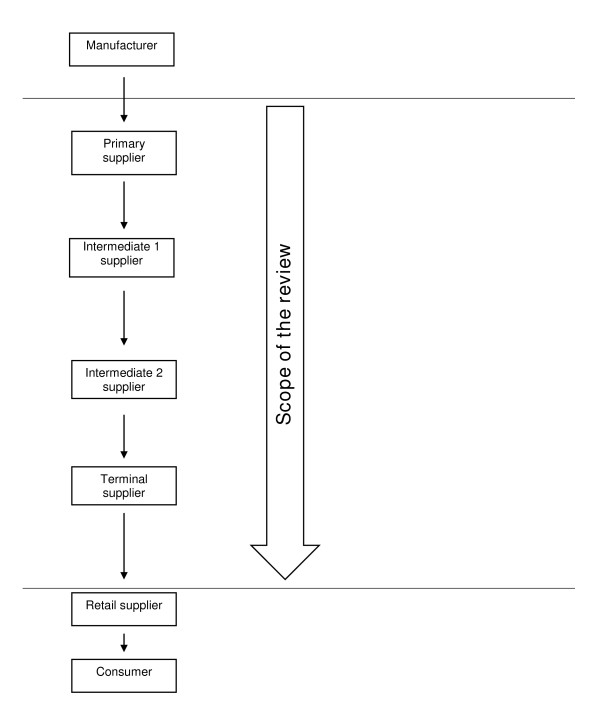
**Taxonomy of suppliers operating within the distribution chain**. Suppliers directly serving retailers are referred to as *terminal *suppliers. They buy from upstream suppliers, referred to as *primary *suppliers if they are the point of entry into the distribution chain after a drug has left the factory gate, or *intermediate *suppliers if they themselves obtain drugs from primary suppliers.

The retail sector distribution chain refers to all levels of the in-country distribution chain, in other words to the chain of wholesalers serving the retail sector. The focus is on suppliers who operate from the point where commodities leave the factory gate or port of entry down to those directly supplying retailers. For the purpose of the review, a taxonomy of suppliers was developed (Figure 1). Suppliers who sell directly to retailers are termed *terminal *suppliers. These buy from upstream suppliers, referred to as *primary *suppliers if they are the point of entry into the distribution chain, or *intermediate *suppliers if they themselves obtain drugs from primary suppliers.

### Literature search and review strategies

The search strategy aimed to identify published, grey and unpublished studies on the retail sector distribution chain for malaria treatment in low- and middle-income countries. Published studies were identified by searching web-based databases, using key terms pertaining to market structure and price mark-ups (Table 1). Grey and unpublished sources were identified by searching the websites of organizations involved in research related to the distribution chain for malaria treatment in low and middle income countries and contacting key informants within these institutions (the William J. Clinton Foundation, Medicines for Malaria Venture, Dalberg Global Development Advisors, Health Action International Europe, MIT-Zaragoza International Logistics Program). Searches were finalized in February 2009.

**Table 1:** Published literature search strategy: databases and key words

Databases	PubMed	EconLit	IBSS
**Key words**	Private sector†; Commerce†*; Private providers; Retail sector; Supply chain; Distribution chain Anti-malarials†; Malaria†; Non-prescription drugs†;Prescription drugs†; Drugs, essential† Price; Pricing; Markup(s); Profit margin; Price component Developing countries†; Africa†; Asia, Western†; Asia, Southeastern†; Latin America†	Private sector; Retail sector; Wholesale; Supply chain; Anti-malarials; Pharmaceuticals Price; Pricing; Markup(s); Profit margin; Price component	

† Mesh term; *The interchange of goods or commodities, especially on a large scale, between different countries or between populations within the same country. It includes trade (the buying, selling, or exchanging of commodities, whether wholesale or retail) and business (the purchase and sale of goods to make a profit) [http://www.ncbi.nlm.nih.gov/sites/entrez, accessed 10 March 2008]. PubMed searches were limited to government publications, journal articles and technical reports.

Studies were included if they provided data specifically on anti-malarials for products stocked, volume sold and mark-ups. Studies that looked at the structure of the distribution chain, in terms of supply sources, supplier numbers and characteristics for both anti-malarials and medicines in general were also included on the basis that anti-malarials are expected to follow the same distribution route as other drugs and represent an important share of all drugs distributed in developing countries.

The review focuses on wholesalers but includes two aspects of retailer behaviour relevant to the study of the distribution chain: their sources of supply and the mark-up they add at the retail level. Other aspects of the retail market, such as its structure and operations have been reviewed elsewhere [24]. Studies were excluded if they compared retail prices to international reference prices without any information on price components across distribution chain levels.

## Results

Thirty-two references exploring the distribution chain for anti-malarials and pharmaceutical drugs in general were identified. The evidence they provide focuses mainly on supply sources, with more limited attention to the number and characteristics of suppliers, and anti-malarial sales volumes and mark-ups (Table 2). Methods used included document reviews and structured or unstructured interviews with retailers, wholesalers and informants working at central government level.

**Table 2:** Overview of the literature

Reference	Distribution chain structure			Anti-malarial products	
	**Source of supply**	**Number of suppliers**	**Suppliers' characteristics**	**Volumes sold**	**Mark-ups**

**Burkina Faso**					

RBM Secretariat, 2007 [77]	X	X	-	-	X

**Cambodia**					

Institute of Medicine, 2004 [48], Shretta and Guimier, 2003 [55]	X	X	-	-	X

PSI, 2008 [58], Sabot, 2009 [63]	X	-	-	X	X

Rozendaal, 2001 [44]	X	-	-	-	-

**Cameroon**					

Van der Geest, 1987 ± [19]	X	-	X	-	-

RBM Secretariat, 2007 [54]	X	X	-	-	X

**Ghana**					

Buabeng et al., 2008 [56]	X	-	-	-	-

**Kenya**					

Marsh et al., 2004 [57]	X	-	-	-	-

Ministry of Health of the Government of the Republic of Kenya, 2004 [78]	-	-	-	-	X

Myhr, 2000 [61]	-	-	-	-	X

Tavrow, 2003 [25]	X	X	X	-	X

Amin and Snow, 2005 [27]	X	-	-	-	-

**Mozambique**					

Russo, 2007 ± [49]	X	X	-	-	-

**Nigeria**					

Adikwu, 1996 ± [17]	X	-	-	-	-

IFC, 2008 ± [52]	X	X	-	-	-

**Senegal**					

Institute of Medicine, 2004 [48], Shretta and Guimier (2003) [55]	X	X	-	X	X

Kone et al., 2007 [62]	X	-	-	-	X

IFC, 2008 ± [52]	X	X	-	-	-

**Sri Lanka**					

Rajakaruna et al., 2006 [47]	X	-	-	-	-

**Tanzania**					

Battersby et al., 2003 [31]	X	X	-	-	X

Goodman, 2004 [20], Chukwujekwu, 2007 [32]	X	X	X	-	X

Clinton Foundation, 2008 [34]	X	-	-	X	X

Government of the Republic of Tanzania and Clinton Foundation, 2008 [29]	X	X	-	X	X

**Uganda**					

Adome et al.,1996 [18]	X	-	-	-	-

The Republic of Uganda, 2004 [59]	X	-	-	-	X

MMV, 2007 [26]	X	X	-	-	X

Yadav and Conesa, 2008 [51]	-	X	-	-	-

IFC, 2008 ± [52]	X	X	-	-	-

RBM Secretariat, 2007 [54]	X	X	-	-	X

**Zambia**					

Institute of Medicine, 2004 [48], Shretta and Guimier, 2003 [55]	X	X	-	-	X

Yadav, 2007 ± [50]	X	X	-	X	-

Clinton Foundation, 2008 [33]	X	-		-	X

**Low/Middle income countries**					

Foster, 1991 ± [13]	X	-	-	-	-

Yadav and Ongola, 2007 ± [53]	X	X	-	X	-

± = studies on distribution chain for pharmaceutical drugs in general (other studies are specific to anti-malarials)

### Structure of the distribution chain

This section summarizes evidence on the shape of the distribution chain and the number and types of suppliers operating at each level. Overall, the chain had a pyramid shape similar to that of other private distribution channels, with fewer suppliers at the top and more numerous suppliers at the bottom [25,26,29,31,32,48-55]. The number of levels within the chain ranged from zero (in the case where retailers obtained drugs directly from the factory gate) up to four levels (in the case of a chain made up of terminal, two intermediate and primary levels). The chain serving more remote outlets and those with less qualified staff tended to have more numerous levels. There were two intermediate levels of general wholesalers in the chain serving general shops operating in three rural districts in Tanzania but no intermediate level in the chain serving drug shops located in the same districts [32]. In a rural district of Uganda, two intermediate levels of wholesalers supplied the chain down to general stores and market stalls whilst the chain serving drug shops had a single intermediate level of wholesalers [26].

Data on the total number of suppliers operating at each level of the anti-malarial distribution chain were generally lacking. When available, data mainly concerned registered suppliers of pharmaceutical products in general [25,26,31,48-50,52] and rarely provided information on the total number of suppliers handling anti-malarials [29,32,51,53]. Overall the number of importers operating in a country was reported to range from 1 to 50 [53]. In Burkina Faso, there were 4 private importers and in Uganda 15 importers and 50 wholesalers, with the latter sometimes owned by importers [54]. The type of businesses acting as terminal, intermediate and primary suppliers is described below, although as will become clear, there is considerable overlap between these categories in practice.

At the terminal level, wholesalers were the most common suppliers, serving pharmacies [25-27,33,48-50,52-54,56], drug shops [26,28,29,31-34,48,50,53,54,57,58] and general shops [25-28,32,33,50,53,57]. In some settings, different types of wholesalers tended to supply different types of retail outlets. In Tanzania and Kenya, wholesalers who supplied drugs alongside other commodities served general shops [25,28,32], whilst wholesalers specialized in handling drugs usually served pharmacies [25,27] and drug shops [28,32].

Retailers themselves frequently operated as terminal suppliers for outlets located in more remote areas [13], although with variation across countries and retailer types. Pharmacies frequently supplied rural drug shops [26,31,48,50] and general stores [17,26,50], sometimes in a relatively organized manner, such as in Nigeria where they sent sales teams [17]. Drug shops were somewhat less common terminal suppliers, at times serving other drug shops in Uganda and Tanzania [26,29] and general stores in Uganda only [26].

Importers were also terminal sources when they directly served pharmacies [26,49,50,56], which they sometimes owned [26,49,50], and also drug shops [26,32,50], using sales teams, such as in Tanzania [32].

Public agencies were terminal suppliers, either officially such as in Sri Lanka where the State Pharmaceutical Corporation supplied retail outlets [47] or unofficially in other countries, where government health workers sold public sector drugs to retail shops, such as in Uganda and Cameroon for example [18,19,48].

Terminal suppliers' characteristics were rarely explored. When available, the evidence shows that in Tanzania wholesalers infrequently had any health-related qualifications, although drug specific wholesalers were reported to employ more qualified staff (mainly pharmacy and biochemistry graduates) and to have been in operation for longer than general wholesalers [32].

Information on terminal suppliers' locations shows that overall, remotely located drug shops and general stores obtained their supplies more locally than more accessible retailers. In Zambia, 24% of outlets located in three border districts with DR Congo or Tanzania obtained their drugs from district suppliers and the same proportion chose to cross borders to buy from Tanzanian or Congolese suppliers [33]. In Tanzania, drug shops generally obtained anti-malarials from drug specific wholesalers or pharmacies located in the capital city, hundreds of kilometres away [28,29,32], whilst those located more than 1,000 kilometres away from the capital city obtained their supplies from more nearby locations [29]. In Uganda and Kenya, general shops usually obtained their supplies from local suppliers [25-27]. In Kenya, the location of general shops' supply sources varied with outlet size, such that large shops where more than one person worked during opening hours obtained their supplies from general wholesalers located inside or outside the district whilst smaller shops where one person worked during opening hours bought more frequently from general wholesalers located within the district [27].

Mobile suppliers, such as sales representatives of drug companies or general distributors, served retailers in many settings, although their popularity and the types of outlets they served varied. In Kenya, mobile vendors commonly supplied both drug and general shops [25,27,57], whilst in Tanzania mobile vendors only served general shops, representing in some districts only 1% of supply sources [28], but in others being a more common source of supply [31]. In Nigeria, sales representatives of large national and international drug companies supplied all types of retail outlets [17]. By contrast, in Uganda and Tanzania, local manufacturers' sales teams supplied the more accessible retailers with more qualified staff [26,32]. Finally, overseas manufacturers directly supplied retailers in Sri Lanka only where 5% of retailers obtained drugs directly from drug companies in India [47].

At intermediate level, studies provided much less information on supply sources. In settings where intermediate-level suppliers were identified [26,32,48,52], they were wholesalers who, as in the case of those operating at terminal level, either handled drugs alongside other commodities or specialized in drugs, hence supplying distinct distribution chains. Information on the location of intermediate suppliers was available only for Tanzania and Uganda, where they operated in the capital city [26,32] and at regional [32] or district level [26]. In Tanzania, intermediate wholesalers were sometimes agents of upstream suppliers at regional level [32]. Regional wholesalers also, at times, used mobile services providing door-to-door services to their customers [32]. In other settings, there was no information available at this level or no intermediate suppliers operating in the chain serving the studied areas [18,25,27,58]. Finally, as at terminal level, information on suppliers' characteristics was provided by a single study reporting that in Tanzania general suppliers had started their business more recently than drug specific wholesalers and rarely employed staff with health related qualifications [32].

At the top of the chain or primary level, suppliers were importers who were agents of overseas pharmaceutical companies, sometimes contracted to act as their sole supplier for distributing their products locally [26,32,50] or, more rarely, integrated with overseas companies as seen in Mozambique [49]. The literature provided little information on the nature of this agency relationship. In the case of exclusive distributorship agreements between overseas companies and local importers, the latter frequently exchanged products with other importers for which one or the other was the sole supplier [26,31,32,49,50], creating horizontal transactions at the top of the chain. This situation was reported in Zambia where importers tended to have regular customers who would generally purchase the bulk of their supplies from few importers. As importers were generally the sole entry point for a particular drug, they would often exchange products between one another [32,50] rather than send customers to buy from the relevant importer. As a result, no clear differentiation between wholesalers and importers existed in many settings, as these roles were product dependent [50,59]. As at terminal and intermediate levels, suppliers' characteristics were provided only by the study conducted in Tanzania, where drug-specific suppliers employed more staff with health-related qualifications and had been in operation for longer than general suppliers [32].

Finally, illegal distribution channels were reported in several countries, whereby drugs were smuggled from one country to another [19,44,48,56]. For example, drugs smuggled from Nigeria were commonly found on sale in Cameroon or passing through Cameroon to reach Gabon or the Central African Republic [19]. In Senegal, smuggling took the form of sea or air shipments diverted from their initial destination or illegal imports of donations from European countries [48]. Whilst illegal channels were commonly reported, the literature offered very limited information on their structure and actual size [19]. In Zambia, illegal importers were found to serve wholesalers and drug shops directly [48].

**Figure 2:**
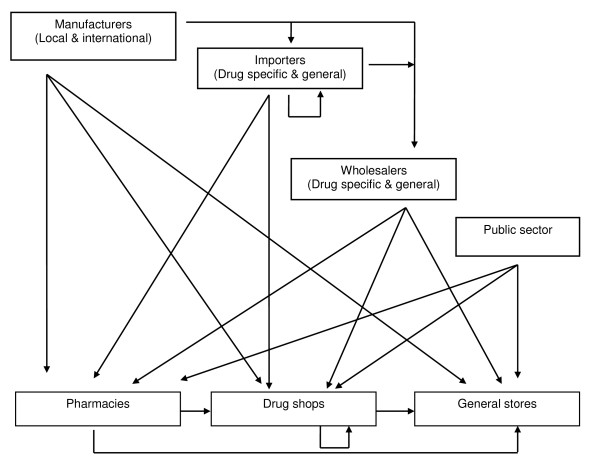
**Structure of the distribution chain**. This figure shows the complexity of the in-country distribution chain for anti-malarial drugs in low and middle-income countries, as reported in the literature.

This section shows that the distribution chain is far more complicated than as characterized in our taxonomy (Figure 1). Figure 2 represents what happens in reality, as reported in the literature.

### Anti-malarial sales volumes and mark-ups

Sales volume estimates are key data for assessing the relative importance of wholesalers within the distribution chain and understanding suppliers' pricing decisions. Data on actual volumes sold across chain levels were found in only six references [29,34,48,53,55,60]. Anti-malarial sales volumes reported by 21 wholesalers operating across six regions of Tanzania ranged from 2,001 and 27,000 doses per month [34]. The rest of the literature indicated relatively concentrated wholesale markets (compared to retail markets), especially at the top of the chain where a few suppliers were responsible for most of the volume sold [33,51,53]. Only one study on the anti-malarial import market in Uganda calculated concentration ratios (the proportion of anti-malarial sales volumes/value accounted for by the n largest firms) and the Hirshman-Herfindahl index (HHI) (the sum of squared market shares of each firm in the market). The study found that five importers accounted for nearly 72% of anti-malarial sales with a HHI of just under 1400, indicating moderate market concentration (an index under 1,000 is associated with competitive markets and above 1,800 with monopoly) [51].

More attention has been paid to measuring anti-malarial price mark-ups, especially on first-line treatments for uncomplicated malaria or the most common alternatives at the time of the studies. Methods used included regulatory document reviews, qualitative interviews with key informants including government officials, wholesalers and retailers [48,54,61], sometimes combined with semi-structured or structured interviews with wholesalers, retailers and/or consumers (Table 3). For the purpose of this review, mark-up data were summarized using a specific taxonomy. Primary mark-ups, therefore, refer to the margins that primary suppliers (entry point to the distribution chain) add on top of their purchase prices when they serve intermediate or terminal wholesalers. Terminal mark-ups relate to margins added by terminal wholesalers (retailers' direct supply sources) on top of the price at which they obtained the drug, either from primary or intermediate suppliers (Table 3).

**Table 3:** Mark ups on anti-malarial drugs

Country	Methods (study reference)	Generic name* (drug type or brand)	Product description, (as provided in the literature)	Mark ups across supply chain levels						
				**Primary**	**Intermediate 1**	**Intermediate 2**	**Terminal**	**Retail (location where available)**		

								**Pharmacies**	**Drug shops**	**General shops**

Burkina Faso	Document review; KII§ Semi-structured interviews with suppliers [54]	CQ	1 dose	**-**	**-**	**-**	**-**	100%	-	-
		
		SP	1 dose	**-**	**-**	**-**	**-**	100%	-	-
		
		ACT	1 dose	**-**	**-**	-	30%	100%	-	-

Cameroon	KII [54]	ACT	1 dose	**-**	**-**	-	14%	34%	**-**	**-**

Cambodia	Semi-structured interviews with suppliers and retailers [48,55]	AS	18 tablets	-	-	-	2%	3%	-	-
		
		AS+M ± (Malarine^®^)	Child dose	-	-	-	50%	3%	-	-
	
	Structured interviews with suppliers [58]	AS+M ± (Malarine^®^)	Adult dose	-	-	-	-	71% **		-
		
		AS+M ± (Malarine^®^)	Child dose	-	-	-	-	65% **		-
		
		AS+M2	8 tablets	-	-	-	-	29% **		-
		
		AS+M3	12 tablets	-	-	-	-	15% **		-
		
		AS+M4	17 tablets	-	-	-	-	16% **		-

Kenya	KII and structured survey of retailers [78]	AQ (IB)	9 tablets	40%	-	-	15%	33%	-	-
		
		SP (IB)	3 tablets	29.5%	-	-	15%	33%	-	-
		
		SP (G, LPG)	3 tablets	-	-	-	15%	203%	-	-
	
	Semi-structured interviews with retailers [25]	AQ (Malaramed^®^)	Child dose, syrup	-	-	-	-	86%	-	-
		
		AQ (Amobin^®^)	Child dose, syrup	-	-	-	-	22.9%	-	-
		
		AQ (Malaratab^®^)	Child dose	-	-	-	-	189%	-	-
		
		SP (Laridox^®^)	Child dose	-	-	-	-	151%	-	-
		
		SP (Fansidar^®^)	Child dose	-	-	-	-	13%	-	-
		
		SP (Falcidin^®^)	Child dose	-	-	-	-	28%	-	-
	
	Document review [61]	AMs	-	-	-	-	15%	20%	-	-
	
	KII [54]	ACT	1 pack	-	-	-	10%	33%	-	-

Senegal	KII; Mystery shopper technique at retail level [62]	AS+AQ ±	Adult dose	-	-	-	15%	3-5%	-	-
			
			Child dose	-	-	-	15%	11-22%	-	-
	
	Semi-structured interviews with suppliers and retailers [55]	Q (IB, BG)	1 dose (injection)	-	-	-	18%	41%		-
		
		Q (G)	1 dose (injection)	-	-	-	15%	30%		-

Tanzania	Semi-structured interviews with suppliers and retailers [20,32]	AQ	1 tablet	-	-	-	9%	-	270%-669% (rural)	**-**
		
		AQ	1 tablet	-	-	8%	-	-	-	-
		
		Q	1 tablet	-	-	-	26%	-	150%-203% (rural)	-
	
	Semi-structured interviews with suppliers and retailers [31]	SP	3 tablets	48%	-	-	13%	-	-	100-233%
	
	Semi-structured interviews with suppliers and retailers [29,30]	AL (IB) ±^i^	5<15 kg dose	-	-	-	67% **	-	100-200%	-
			
			15<25 kg dose	-	-	-	56% **	-	60%-221%	-
			
			25<35 kg dose	-	-	-	52% **	-	47%-230%	-
			
			35+ kg dose	-	-	-	50% **	-	39%-233%	-
		
		AL (IB) ±^ii^	5 <15 kg dose	43%	-	-	-	-	100-200%	-
			
			15<25 kg dose	34%	-	-	-	-	60%-221%	-
			
			25<35 kg dose	31%	-	-	-	-	47%-230%	-
			
			35+ kg dose	27-30% **	-	-	-	-	39%-233%	-
	
	Semi-structured interviews with suppliers and retailers [34]	ACT (IB)	n/a†	-	-	-	21%	-	54% (rural)	-
		
		AMT(IB)	n/a†	-	-	-	18%	-	44% (rural)	-
		
		SP	n/a†	-	-	-	23%	-	110% (rural)	-
		
		AQ	n/a†	-	-	-	41%	-	96% (rural)	-
		
		Quinine	n/a†	-	-	-	38%	-	64% (rural)	-

Uganda	Semi-structured interviews with suppliers and retailers [59]	SP (MSG)	3 tablets	-	-	6%	-	410%	-	-
		
		SP (LPG)	3 tablets	27%	-	-	29%	501%	-	-
	
	KII; Semi-structured interviews with retailers [54]	All AMs	n/a	40-50%	-	-	7-8%	-	-	-
		
		AL (G)	1 dose	-	-	-	-	38%		-
		
		CQ (G)	1 dose	-	-	-	-	100%		-
	
	Semi-structured interviews with suppliers and retailers [26]	DHA+PP (IB)	1 tablet	32%	-	-	14%	-	29% (rural)	-
		
		DHA+PP (IB)	1 tablet	32%	-	-	21%	22% (rural)	-	-
		
		SP (IB)	1 tablet	57%	-	-	8%	-	43% (rural)	-
		
		SP (IB)	1 tablet	57%	-	-	16%	50%(rural)	-	-
		
		SP (G)	1 tablet	-	-	-	40%	-	198% (rural)	-
		
		SP (G)	1 tablet	-	-	-	13%	271%(rural)	-	-
		
		CQ (G)	1 tablet	-	-	-	18%	152%(rural)	-	-
		
		Artemether (IB)	1 ampoule	99%	-	-	33%	-	50% (rural)	-
		
		Artemether (IB)	1 ampoule	56%	-	-	16%	28%(rural)	-	-
		
		SP (G)	1 tablet	-	-	-	25%	-	200% (rural)	-
		
		CQ (G)	1 tablet	-	-	-	41%	-	92% (rural)	-
		
		DHA+PP (IB)	1 tablet	36%	-	-	11%	65%(urban)	-	-
		
		Artemether	1 ampoule	56%	-	-	17%	-	136% (urban)	-
		
		Artemether	1 ampoule	56%	-	-	17%	82%(urban)	-	-
		
		SP (IB)	1 tablet	57%	-	-	5%	85%(urban)	-	-
		
		SP (G)	1 tablet	-	-	-	25%	566% (urban)	-	-
		
		CQ (G)	1 tablet	-	-	-	24%	143% (urban)	-	-

Zambia	Structured interviews with suppliers [33]	ACT	-	-	-	-	-	-	60%	-
		
		SP	-	-	-	-	-	182%	-	-
		
		ACT	-	-	-	-	-	29%, 11%-100% (urban)		
		
		ACT	-	-	-	-	-	67%,13%-100% (peri-urban)		
		
		ACT	-	-	-	-	-	54%, 50-100% (rural)		
		
		SP	-	-	-	-	-	50%, 15%-327% (urban)		
		
		SP	-	-	-	-	-	300%, 50%-517% (peri-urban)		
		
		SP	-	-	-	-	-	50%, 15%-500% (rural)		
	
	Semi-structured interviews with suppliers and retailers [48,55]	Selected AM ¥	-	-	-	-	-	30%		

* AM = anti-malarials; AMT = artemisinin monotherapies; mg = milligrams; ml = millilitres; AS = Artesunate; M = Mefloquine; AS+M2 = combination for children weighing between 16 kgs to 24 kgs; AS+M3 = combination for children weighing between 25 kgs to 35 kgs; AS+M4 = combination for adults; AQ = Amodiaquine; SP = Sulphadoxine-Pyrimethamine; Q = Quinine; AL = Artemether-Lumefantrine; DHA+PP = Dihydroartemisinin+Piperaquine; IB = imported innovator brand, IG = imported generic, B = branded; G = locally produced generic, MSG = most sold generic, LPG = lowest priced generic, BG = branded generic; I = imported, LP = locally produced; SC = supply chain; ± = subsidized product; - = level of the chain did not exist or data not available; **Author's own calculations; † mean across all products within drug class. ^i ^primary supplier is the terminal supplier, ^ii ^primary supplier sells to terminal regional supplier. ¥ included AQ (3 tablets), Artemether (not stated), AS (6 tablets), CQ (1000 tablets), DHA (not stated), Halofantrine (6 tablets), Mefloquine (3 tablets), Proguanil (not stated), Q (1000 tablets), AL IB (6 tablets), SP (3 tablets). IFC = International Finance Corporation. Mark-up data were rounded to the nearest whole number. §KII = key informant interviews; Mystery shopper technique = unobtrusive observation of shop attendants by researchers who pose as client seeking care from a provider who is unaware of their identity.

Overall, studies reported mark-ups within the distribution chain serving pharmacies or/and drug shops, except one that also provided anti-malarial mark ups within the chain supplying general stores.

Within the distribution chain, mark-ups varied across levels, ranging from 27% to 99% at primary level, 8% at intermediate and 2% to 67% at terminal level (Table 3). In some settings, mark ups varied depending on the structure of the chain [26], with somewhat higher mark-ups at a given level observed in a distribution chain made of fewer levels. For example, in Tanzania, when supplying regional wholesalers, importers added between 27% and 43%, whilst when directly supplying retailers they added between 50% and 67% [29].

In the retail market, price mark-ups on anti-malarials have been relatively more researched. They were sometimes very high and varied greatly across outlet type and location, and anti-malarial type and packaging. There were four key findings. First, mark-ups ranged between 3% and 566% in pharmacies, 29% and 669% in drug shops and 100% and 233% in general shops (Table 3). Second, mark-ups were somewhat higher in rural outlets compared to urban ones. In Zambia, for example, the median ACT mark-up in Lundazi, a rural district was 54% whilst in Kabwe urban district the median was 29% [33]. In Choma, a peri urban district, the median ACT mark-up was, however, much higher than in rural Lundazi reaching 300% [33]. Third, generics tended to have higher percentage mark-ups, a situation that may not have translated into higher absolute margins given that generics are generally sold at lower prices than branded products. Fourth, mark-ups varied across packaging types, with a mark-up of 669% on one loose tablet of amodiaquine compared to 270% on a blistered tablet in Tanzania [20]. Again, assuming that loose tablet prices are lower than packed tablet prices, this may not have automatically translated into higher absolute margins.

In some settings, where ACT subsidy schemes have already been implemented, mark ups were within the range expected by the managers of the schemes. In Senegal, private pharmacies purchased the subsidized first-line ACT from public sector medical stores and added on average 35% to the price of an adult dose, which translated into a retail price only 4% higher than the recommended retail price (RRP) [60,62]. In two districts of Tanzania, a subsidy scheme was piloted in drug shops and in one of these two districts, it was combined with a RRP printed on ACT packs. ACT availability increased and the subsidy effectively decreased the price of ACT below the price paid by consumers in the control area and below the price of older anti-malarials, leading to a large increase in the proportion of anti-malarial consumers purchasing ACT in the two intervention areas (from 1% to 44.2% one year later) [63]. Surprisingly, ACT prices were higher in the district with the RRP than in the district without, suggesting caution in future use of this approach for controlling ACT retail prices [63]. In Cambodia, a contrasting experience of a subsidy scheme was reported. Cambodia is the first country to have switched its first-line treatment to ACT and implemented a social marketing programme, including a subsidy, packs printed with RRP and mass communication campaigns in its endemic provinces. Market penetration of the subsidized ACT remained relatively low and ACT retailed, on average, at a price 70% higher than the RRP [60].

Overall, relatively little is known about the factors that influence pricing decisions. Only one study was identified which used multivariate statistical methods to analyse price determinants, examining prices in drug and general retail shops selling anti-malarials in rural Tanzania. The study found that higher retail prices were associated with branded and packed products, being sold in general shops (which might have reflected higher prices charged by their terminal supply sources) and higher market concentration [16,20]. The rest of the literature provided descriptive findings. Retail and wholesale margins were reported to be influenced by fixed price or margin regulation or, in the absence of regulation, market competition and consumer demand. Wholesale pricing decisions were also reported to be influenced by product characteristics, business practices and costs [26,32,54]. In Uganda, markups were reported to be lower for anti-malarials with shorter shelf life [26]. In Tanzania, drug wholesalers reported giving discounts to customers who bought drugs in relatively large quantities [32], and general wholesalers to customers who purchased drugs alongside other commodities [32]. One wholesaler also reported adding 6-7% to cover his expenses and 3-4% for profit [32].

## Discussion

The existing evidence on the retail sector distribution chain for anti-malarial drugs was reviewed by identifying 32 references across 12 low and middle-income countries. The distribution chain has a pyramid shape and its structure varies greatly across countries and within countries across outlet types, with chains having more levels when serving rural and less formal outlets. There was also some indication of weak competition especially at primary level, where few wholesalers accounted for most of the anti-malarial volumes sold. Wholesale mark-ups were lower than retail mark-ups and these varied across chain levels and anti-malarial drug types.

Overall, there was a lack of representative data on a national scale, which made the interpretation of data difficult. Studies tended to focus on the distribution chain serving a single type of outlet, often the more formal type, such as pharmacies generally operating in urbanized settings. Data on the number of wholesalers who operate across levels was restricted to registered businesses and information on their characteristics was generally lacking. Studies were mainly descriptive and provided limited evidence on the influence of the distribution chain on retail anti-malarial availability and prices. Sales volume data across chain levels were nonexistent and mark-up data were concentrated at retail and terminal levels, with less information at primary and particularly intermediate levels (one study only). This situation can be explained by the methods that have been used to study key variables, which were often limited to document reviews and interviews with key informants (central government, industry representatives) or retailers. Evidence on stocking and pricing decisions within the distribution chain was therefore lacking, an important knowledge gap for improving consumers' access to affordable quality malaria treatment. High mark-ups and prices are commonly perceived as a sign of high profit, often leading to calls for medicine price reduction [64]. However, without information that disaggregates mark-ups into profits and costs it is unclear if such measures are appropriate.

A strong interest in working with retailers to improve the quality of care they provide has emerged in recent years. Goodman and colleagues identified 16 interventions working with medicine sellers to improve malaria treatment, all including a mix of activities such as training and capacity building, demand generation, quality assurance and creation of an enabling environment [28]. However, only two of the 16 interventions were implemented within the distribution chain, involving training wholesalers and mobile vendors in Kenya and sales representatives in Madagascar. Whilst the evidence available on the outcomes of these initiatives was weak and particularly limited in terms of the sustainability and equity of benefits, it showed some improvements in retailers' knowledge and/or performance [28].

The Affordable Medicine Facility for malaria (AMFm) [65] aims to increase coverage of effective treatment and delay the development of drug resistance, by subsidizing ACT at the top of the distribution chain and implementing supporting interventions such as training, regulatory strengthening and consumer education. The capacity of AMFm to meet its goals has been extensively debated [66-68], including how the structure of the distribution chain and nature of competition at all levels will affect final prices. Sceptics are concerned that the subsidy will be captured by middle-men within the private commercial supply chain and informal unqualified profit-maximizing retailers. This review indicates that there is insufficient evidence on anti-malarial distribution chains to predict with confidence what the outcome will be, particularly reflecting inadequate information on profit margins and the factors that influence pricing decisions. On the one hand, relatively concentrated markets (few suppliers accounting for large share of sales) were documented at the primary supplier level in Uganda and Zambia, accompanied by frequent exclusive dealership relationships, and within local areas at retail level, indicating the potential for exploitation of market power. On the other hand, early experiences of subsidizing ACT provide valuable lessons, notably the importance of rigorous distribution chain analysis, for example to set the RRP at an appropriate level. Reducing the price of ACT will however not suffice and accompanying interventions need to be identified and tailored to each country context [69]. For example, Rapid Diagnostic Tests (RDTs) have the potential to increase access to accurate diagnosis and appropriate treatment, especially in remote areas where alternative routine microscopy services cannot easily be made available [70-73]. However, the distribution of affordable quality RDTs is also not without challenges [71,74,75] and has been the object of little research to date [76].

## Conclusions

Available evidence on the distribution chain for retail sector malaria treatment provides some useful descriptive information, but there is a lack of nationally representative data, and of analysis of the determinants of supplier behaviour. In the advent of the AMFm, a better understanding of the role of the anti-malarial distribution chain on retail outcomes is urgently needed. Retailers are likely to remain an important source of malaria treatment and the knowledge gaps identified here could jeopardize the success of initiatives for improving ACT access. Addressing these uncertainties should be a priority of ongoing and future research.

## Competing interests

The authors declare that they have no competing interests.

## Authors' contributions

EP, CG and KH developed the search strategy. EP conducted the search strategy, reviewed the literature and drafted the manuscript. CG helped to draft the manuscript. CG and KH critically revised the manuscript. All authors read and approved the final manuscript.
